# Myelodysplastic syndromes with del(5q): A real-life study of determinants of long-term outcomes and response to lenalidomide

**DOI:** 10.1038/s41408-022-00724-3

**Published:** 2022-09-07

**Authors:** Carmelo Gurnari, Alfonso Piciocchi, Stefano Soddu, Fabrizio Bonanni, Emilia Scalzulli, Pasquale Niscola, Ambra Di Veroli, Anna Lina Piccioni, Monica Piedimonte, Gianluca Maiorana, Prassede Salutari, Laura Cicconi, Michelina Santopietro, Svitlana Gumenyuk, Chiara Sarlo, Susanna Fenu, Agostino Tafuri, Roberto Latagliata, Luana Fianchi, Marianna Criscuolo, Jaroslaw P. Maciejewski, Luca Maurillo, Francesco Buccisano, Massimo Breccia, Maria Teresa Voso

**Affiliations:** 1 Department of Biomedicine and Prevention, University of Rome Tor Vergata, Rome, Italy; 2 Department of Translational Hematology and Oncology Research, Taussig Cancer Institute, Cleveland Clinic, Cleveland, OH USA; 3 GROM-L (Gruppo Romano-Laziale MDS), Rome, Italy; 4 Italian Group for Adult Hematologic Diseases (GIMEMA) Foundation, Rome, Italy; 5 Department of Translational and Precision Medicine-Az., Policlinico Umberto I-Sapienza University, Rome, Italy; 6 Hematology Unit, Sant’Eugenio Hospital, Rome, Italy; 7 Belcolle Hospital, Viterbo, Italy; 8 Hematology Dep, Az. Osp., San Giovanni-Addolorata, Rome, Italy; 9 Hematology, “Sant’Andrea” Hospital-Sapienza, University of Rome, Rome, Italy; 10 Hematology, Pescara Hospital, Pescara, Italy; 11 Hematology, ASL Roma 1, Rome, Italy; 12 Hematology and Hematopoietic Stem Cells Transplant Unit, AO San Camillo-Forlanini, Rome, Italy; 13 Hematology Unit, Department of Research and Clinical Oncology, IRCCS Regina Elena National Cancer Institute, Rome, Italy; 14 Hematology and Stem Cell Transplantation Unit, University Campus Bio-Medico, Rome, Italy; 15 Department of Hematology, Fondazione Policlinico Universitario Agostino Gemelli-IRCCS, Roma, Italy; 16 Santa Lucia Foundation, IRCCS, Neuro-Oncohematology, Rome, Italy

**Keywords:** Risk factors, Myelodysplastic syndrome

**To the Editor:**

Deletion of the long arm of chromosome 5, namely del(5q), is among the most recurrent cytogenetic aberrations in myelodysplastic syndromes (MDS), accounting for approximately 10–15% of all cases. Del(5q) defines a unique MDS sub-category, and represents the first genomic alteration included in the World Health Organization classification of MDS [1].

Lenalidomide is the first targeted treatment in MDS, being able to abrogate red blood cells (RBCs) transfusion requirements, hallmark of the disease [2]. Patients harbouring del(5q) exhibit an exquisite sensitivity to this agent, which derives its therapeutic window from specifically targeting the haploinsufficient CK1α protein, in addition to displaying indirect immunomodulatory effects and upregulating other disease-associated pathways [1]. While clinical trials have shown erythroid responses in up to two-thirds of patients resulting in improved outcomes, specific predictors of long-term response and survival have not been clearly established [3]. Furthermore, data present in the literature are typically derived from clinical trials conducted in the first decade of 2000, case series with limited median follow-up times (<4-year), or focusing on specific outcomes, *e.g*. leukemia progression [4]. The interest on this latter aspect stems from concerns raised as to the possible leukemogenic potential of lenalidomide, paralleled by worrisome data on increased risk of solid malignancies in long-term survivors of multiple myeloma, the other setting where this drug is routinely used [5].

Here, we took advantage of a large, real-life cohort of del(5q) MDS enrolled in the GROM-L (Gruppo Romano-Laziale MDS) observational study between 2002 and 2021 to unravel clinical determinants of response to lenalidomide and long-term outcomes.

In all cases, conventional metaphase cytogenetics identified interstitial deletion of the long arm of chromosome 5 as previously defined [6]. Patients’ characteristics were summarized by cross-tabulations for categorical variables or by quantiles for continuous variables. Based on the prolonged follow-up reported for the MDS-003 study [7] and the median time under treatment in our cohort, response to lenalidomide was defined as long-term if sustained for at least 36 months, and response criteria were defined according to Cheson et al. [8]. Overall survival (OS, defined as the time from diagnosis to last follow-up or death for any cause) and progression-free survival (PFS, time from the diagnosis to disease progression or death from MDS) estimates were calculated using Kaplan–Meier method. Univariate and multivariate analyses on OS and PFS were performed using Cox regression models, after assessment of proportionality of hazards. Hazard Ratios (HR) and 95% Confidence Intervals were reported as parameter results of the Cox regression models. All covariates were evaluated in univariate models and all factors with univariate association within *p*-value < 0.15 were considered in the multivariate models [9]. All statistical tests were two-sided, and a *p*-value < 0.05 was considered statistically significant. Analyses and data visualization were generated using the computing environment R (4.0.0 R Core Team). The study was approved by the Ethical Committees of participating centers.

A total of 106 patients with del(5q) MDS treated with lenalidomide were enrolled in this study. Because of the absence of follow-up data, 7 cases were excluded from further analysis (Fig. 1A).

**Fig. 1:**
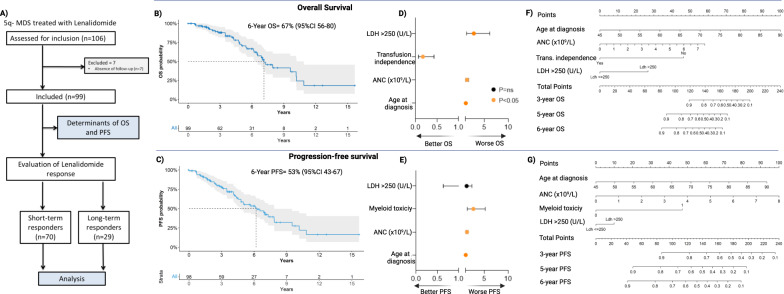
Study design and patients’ outcomes. **A** CONSORT diagram showcases the study design and patient enrollment. **B** A Kaplan-Meier curve shows overall survival (OS) defined as the time from diagnosis of MDS to death for any cause. Censoring was applied to cases alive at the last follow-up. With a median follow-up time of 5.6 years (1.9–6.9), the 6-year OS was 67% (56–80). Numbers at risk are indicated below the curve. **C** A Kaplan-Meier curve shows progression-free survival (PFS) defined as the time from diagnosis to disease progression or death from MDS. Censoring was applied to cases alive at last follow-up. The 6-year PFS was 53% (43–67). Numbers at risk are indicated below the curve. **D**, **E**, Forrest plots illustrate the results of multivariate analysis for OS and PFS, respectively, using a multivariate cause-specific Cox model. Nomograms for OS and PFS are shown in **F** and **G**, respectively. Briefly, each predictor with a given value can be mapped to the Points axis. The sum of these points can be referred to in the Total Points axis. The linear predictor and the probability of survival outcome at 3-, 5-, and 6-year can be obtained from the corresponding axis.

Overall, patients had a median age at diagnosis of 73 years (range, 47–89) and majority were females (M:F = 0.29, Table 1). Anemia was present in all patients at MDS diagnosis, with the majority (>90%) requiring RBCs transfusion support (50% ≥4 units/8 weeks). Absolute neutropenia (<1 × 10^9^/L), and thrombocytopenia (<100 × 10^9^/L) were instead present in only 10 and 12% of cases, respectively. According to the peculiar clinical picture of del(5q) MDS, thrombocytosis (>450 × 10^9^/L)was noticed in 20% of patients, del(5q) was the sole cytogenetic abnormality in 91% of cases, and megakaryocytic dysplasia was detected in 80% of bone marrow (BM) evaluations. Additional cytogenetic alterations included del(20q) and trisomy 8 (*n* = 2 each), del(1p), del(13q), trisomy 12, del(2p) and del(12p). Based on IPSS-R, virtually all MDS (96%) are grouped into very-low to intermediate-risk categories, as also outlined by the low number of BM blasts (median 2%). Increased lactate dehydrogenase (LDH, > 250 U/L) and erythropoietin (≥200 mU/mL) were found in 48 and 39% of patients, respectively, whereas the majority had creatinine levels within normal range (<1.3 mg/dl; 92%).

**Table 1:** Patients’ characteristics at baseline and after lenalidomide treatment.

Characteristics	*N* = 99
Baseline variables	
Gender, *n* (%)	
Male	22 (22%)
Female	77 (78%)
Age*	73 (47–89)
Hemoglobin (gr/dl)*	8.50 (6.50–12.30)
Absolute neutrophil counts (10^9^/L)*	2.00 (0.20–7.80)
Platelets (x10^9^/L)*	305 (28–1,411)
Bone marrow blasts (%)*	2.00 (0–19)
Erythroid Dysplasia, *n* (%)	91 (97%)
Megakaryocytic Dysplasia, *n* (%)	75 (80%)
5q isolated, *n* (%)	87 (91%)
IPSS-R score^^^, *n* (%)	
Very Low	2 (2.4%)
Low	47 (57%)
Intermediate	30 (37%)
High	3 (3.6%)
WHO 2016 diagnosis^&^, *n* (%)	
MDS with isolated del(5q)	89 (89.9%)
MDS-EB-1	8 (8.1%)
MDS-EB-2	2 (2.0%)
Erythropoietin (mU/mL)*	186 (10–941)
Mean corpuscular volume (fL)*	107 (75–226)
Lactate dehydrogenase (U/L)*	275 (139–673)
Creatinine (mg/dL)*	0.90 (0.40–2.20)
Red Blood Cells Transfusion burden, *n* (%)	
<4 units/8 weeks	49 (50%)
≥4 units/8 weeks	49 (50%)
Post-treatment variables	
Cytogenetic Response, *n* (%)	
No response	16 (21%)
Partial	19 (26%)
Complete	39 (53%)
Erythroid Response, *n* (%)	84 (85%)
Transfusion independence, *n* (%)	77 (80%)
Neutropenia (<1 × 10^9^/L) during first 2 cycles, *n* (%)	47 (51%)
Thrombocytopenia (<100 × 10^9^/L) during first 2 cycles, *n* (%)	30 (33%)
Total N. cycles of Lenalidomide*	21 (2–131)
Secondary solid tumor, *n* (%)	6 (6.1%)

^*^median (range); ^^^according to Greenberg et al., Blood 2012; ^&^according to Arber et al., Blood 2016.

Following ESA failure, patients received lenalidomide at a starting dose of 10 mg/day for 21 days/Q28 for a median of 21 cycles (2–131). The median time from MDS onset to treatment start was 10.3 months (0.2–89). Hematological and cytogenetic responses were registered in 84 and 79% of cases respectively, with 80% achieving transfusion-independence for ≥8 weeks. The occurrence of absolute thrombocytopenia (33%) or neutropenia (51%) during the first 2 cycles required dose adjustments in 45% of cases. With a median follow-up of 5.6 years (1.9–6.9), the 6-year OS and PFS were 67% (56–80) and 53% (43–67; Fig. 1B, C), respectively.

Overall, median response duration to lenalidomide was 32 months (24–43; Fig. S1). The occurrence of absolute thrombocytopenia during first cycles was the only variable associated with reduced odds of long-term lenalidomide response (OR = 0.10, 95% CI 0.01–0.36; p = 0.003, Table S1) in univariate setting, whereas no variable was found significant in multivariate analysis.

Older age at MDS onset (HR = 1.09, 95% CI 1.04–1.14; *p* < 0.001), higher neutrophil counts (HR = 1.34, 1.07–1.69; *p* = 0.011) and increased LDH levels (HR = 2.77, 1.25–6.14; *p* = 0.012) were independent predictors of poor OS, whereas the achievement of transfusion-independence was associated with better outcomes (HR = 0.17, 0.07–0.43; *p* < 0.001; Fig. 1D, Table S2). Similarly, older age (HR = 1.04, 95% CI 1.01–1.08; *p* = 0.010) and higher neutrophil counts at baseline (HR = 1.30, 1.08–1.55; *p* = 0.007), along with the occurrence of myeloid toxicity during first cycles (HR = 2.66, 1.36–5.18; *p* = 0.003), were independently associated with worse PFS (Fig. 1E, Table S3). Using the significant prognostic factors of OS and PFS in the Cox-regression model, we built nomograms able to predict the 3-,5-, and 6-year OS and PFS rates (Fig. 1F, G).

Of note, at last follow-up, solid malignancies were registered in 6% of our cohort (adenocarcinomas of colon, two cases, and one case each for stomach, ovary, pancreas, and lung cancer), whereas leukemia progression occurred in 31% of patients at a median of 4.1 years (0.7–10.8) from diagnosis. Apart from a trend for worse OS (*p* = 0.08), patients who subsequently progressed to leukemia did not differ from their counterparts with regards to long-term response rates nor baseline characteristics.

Lenalidomide is now routinely used for patients with del(5q) MDS. Taking advantage of our collaborative group, active in 13 different centers of the Lazio region, we show here that baseline clinical parameters are able to predict long-term outcomes (6-year period) in a real-life setting. In line with previous studies [4, 7], older age at disease onset and higher neutrophil counts were predictors of decreased OS and PFS. Notably, higher neutrophil counts at disease onset have been recently associated with risk of secondary leukemic progression also in bone marrow failure syndromes, probably as a result of survival advantages of clones manifesting with phenomena of age-related myeloid skewing [10]. Increased baseline LDH levels, a marker of severity of ineffective erythropoiesis in lower-risk MDS [11], also affected OS, whereas the early occurrence of lenalidomide-induced myeloid toxicity impacted on PFS. While specific variations in neutrophil and platelet counts during the first 8 weeks of therapy have been reported as a favourable predictor for short-term response [12], the occurrence of cytopenias has been linked with increased risk for leukemic progression [7]. In our experience, the only parameter associated with reduced odds of long-term response was the occurrence of thrombocytopenia during the first 2 cycles, although not confirmed in the multivariate analysis [12]. A baseline platelet count below 280 × 10^9^/L was the strongest predictor for lenalidomide treatment failure in another study [13]. These data indicate that patients with lower platelet counts at disease onset, or developing absolute thrombocytopenia during the first cycles, may be at higher risk of suboptimal response duration, and are candidates for careful follow-up for alternative treatments, including allogeneic stem cell transplantation in younger patients.

The differences in the consideration of cytopenias as statistical variables (absolute cytopenia vs. relative decrease, and the time from lenalidomide start) might explain the discrepancy observed between the current and previous studies focusing on predictors of short-term (8 vs. 26 weeks) response [4, 7, 12, 13]. In line with previous evidence, the achievement of transfusion-independence was associated with significantly improved OS [1, 2, 7].

Leukemic progression rate was in line with prior experience reporting a 5-year cumulative incidence of 40% [7, 14]. A positive safety signal as to the 6% incidence of solid malignancies can be derived from our data, given the prolonged follow-up time and the number of events registered when considering the median age of our cohort.

Currently, no guidelines as to the precise duration of treatment, timing, and modalities of lenalidomide response assessment and, particularly, drug cessation exist [15]. This topic has been gaining attention because of the report of patients transfusion-independent for up to 7-year periods following achievement of complete cytogenetic remission [16, 17]. Besides, borrowing the recent evidence in chronic myeloid leukemia (CML), the deep understanding of del(5q) pathobiology may open possibilities for the application of treatment-free remission strategies also in this setting. In our cohort, no information on treatment-free remission can be provided since lenalidomide was well-tolerated, dose adjustment did not affect long-term outcomes, and no patient discontinued the treatment for reasons other than disease progression or death.

Finally, it is worth mentioning that *TP53* mutations have been found in up to 20% of patients with del(5q) MDS, are associated with increased risk for leukemic progression, but do not prevent initial erythroid or cytogenetic responses [18, 19]. Beyond *TP53*, the occurrence of mutations in other myeloid drivers associated with leukemia progression [14] calls for prospective studies investigating longitudinal clonal dynamics under lenalidomide treatment, possibly paving the way for personalized disease-monitoring approaches.

In conclusion, our long-term, real-life data confirm the efficacy of lenalidomide treatment in del(5q) MDS observed in the setting of clinical trials, and the association with prolonged survival when compared to the pre-lenalidomide era, where a median survival of 57.8 months was reported [20]. We also demonstrate that simple, baseline clinical variables can be used to predict long-term outcomes, although there is an urgent need for studies including molecular predictors. Indeed, the knowledge of the molecular pathogenesis of the disease, the availability of a targeted treatment and the acquired clinical experience, make del(5q) MDS a paradigmatic example, serving as a platform for the introduction of concepts like minimal-residual disease strategies and treatment-free remission in the MDS world.

## Supplementary information

Supplementary Material

## Data Availability

All data are provided in the main text, supplemental material, tables/figures and corresponding legends. Request for additional information may be done via email to the corresponding author Prof. Maria Teresa Voso (voso@med.uniroma2.it).
