# Supplementary material for: Myelodysplastic syndromes with del(5q): A real-life study of determinants of long-term outcomes and response to lenalidomide
Source: Blood Cancer J. 2022 Sep 7;12(9):132. doi: 10.1038/s41408-022-00724-3 (PMC9452671; doi:10.1038/s41408-022-00724-3)
Supplement: Supplementary file 1 — Supplementary Material [file 41408_2022_724_MOESM1_ESM.docx]

**Supplementary Appendix**

**Table S1. Univariate analysis for lenalidomide response at 36 months**

| Characteristic | OR^1^ | 95% CI^1^ | p-value |
| --- | --- | --- | --- |
| **Baseline variables** | | | |
| **Gender** |  |  |  |
| Male | — | — |  |
| Female | 1.14 | 0.41, 3.50 | 0.81 |
| **Age** | 0.97 | 0.93, 1.02 | 0.21 |
| **Hemoglobin (gr/dl)** | 1.24 | 0.85, 1.82 | 0.26 |
| **Absolute neutrophil counts (x10^9^/L)** | 0.96 | 0.73, 1.25 | 0.79 |
| **Platelets (x10^9^/L)** | 0.40 | 0.03, 3.51 | 0.45 |
| **Bone marrow blasts (%)** | 0.89 | 0.67, 1.07 | 0.32 |
| **Megakaryocytic Dysplasia** | 0.50 | 0.18, 1.46 | 0.19 |
| **5q isolated** | 3.41 | 0.58, 64.9 | 0.26 |
| **IPSS-R score(1)** |  |  |  |
| Very Low/Low | — | — |  |
| Intermediate/High | 0.55 | 0.20, 1.44 | 0.24 |
| **WHO 2016 diagnosis(2)** |  |  |  |
| MDS with isolated del(5q) | — | — |  |
| MDS-EB-1/MDS-EB-2 | 0.57 | 0.08, 2.48 | 0.50 |
| **Erythropoietin (mU/mL)** | 1.00 | 1.00, 1.00 | 0.48 |
| **Mean corpuscular volume (fL)** | 1.01 | 0.98, 1.05 | 0.44 |
| **Lactate dehydrogenase (U/L)** | 1.03 | 0.99, 1.07 | 0.21 |
| **Creatinine (mg/dL)** | 0.36 | 0.06, 1.68 | 0.23 |
| **Red Blood Cells Transfusion burden** |  |  |  |
| <4 units/8 weeks | — | — |  |
| >4 units/8 weeks | 0.67 | 0.27, 1.61 | 0.37 |
| **Post-treatment variables** | | | |
| **Cytogenetic Response(3)** |  |  |  |
| No response | — | — |  |
| Partial | 1.75 | 0.41, 8.20 | 0.45 |
| Complete | 1.68 | 0.48, 6.91 | 0.44 |
| **Transfusion independence** | 4.09 | 1.06, 27.0 | 0.073 |
| **Neutropenia (< 1x10^9^/L) during first 2 cycles** | 0.42 | 0.16, 1.03 | 0.064 |
| **Thrombocytopenia (< 100x10^9^/L) during first 2 cycles** | 0.10 | 0.01, 0.36 | **0.003** |
| **Lenalidomide dose** | 0.96 | 0.79, 1.19 | 0.71 |
| ^1^OR = Odds Ratio, CI = Confidence Interval | | | |

**Table S2. Univariate analysis for overall survival**

| Characteristic | HR^1^ | 95% CI^1^ | p-value |
| --- | --- | --- | --- |
| **Baseline variables** | | | |
| **Gender** |  |  |  |
| Male | — | — |  |
| Female | 0.69 | 0.31, 1.53 | 0.36 |
| **Age** | 1.08 | 1.03, 1.12 | **<0.001** |
| **Hemoglobin (gr/dl)** | 0.85 | 0.64, 1.13 | 0.26 |
| **Absolute neutrophil counts (x10^9^/L)** | 1.28 | 1.05, 1.56 | **0.016** |
| **Platelets (x10^9^/L)** | 1.00 | 1.00, 1.00 | 0.33 |
| **Bone marrow blasts (%)** | 1.07 | 0.94, 1.21 | 0.29 |
| **Megakaryocytic Dysplasia** | 0.80 | 0.36, 1.78 | 0.58 |
| **5q isolated** | 2.48 | 0.33, 18.5 | 0.38 |
| **IPSS-R score(1)** |  |  |  |
| Very Low/Low | — | — |  |
| Intermediate/High | 1.15 | 0.55, 2.40 | 0.71 |
| **WHO 2016 diagnosis(2)** |  |  |  |
| MDS with isolated del(5q) | — | — |  |
| MDS-EB-1/MDS-EB-2 | 1.59 | 0.61, 4.14 | 0.34 |
| **Erythropoietin (mU/mL)** | 1.00 | 1.00, 1.00 | 0.16 |
| **Mean corpuscular volume (fL)** | 1.01 | 0.99, 1.03 | 0.21 |
| **Lactate dehydrogenase (U/L)** | 1.00 | 1.00, 1.00 | 0.086 |
| **Creatinine (mg/dL)** | 2.71 | 0.94, 7.86 | 0.066 |
| **Red Blood Cells Transfusion burden** |  |  |  |
| <4 units/8 weeks | — | — |  |
| >4 units/8 weeks | 2.01 | 0.97, 4.15 | 0.059 |
| **Post-treatment variables** | | | |
| **Cytogenetic Response(3)** |  |  |  |
| No response | — | — |  |
| Partial | 0.35 | 0.13, 0.91 | **0.031** |
| Complete | 0.22 | 0.09, 0.57 | **0.002** |
| **Transfusion independence** | 0.19 | 0.09, 0.44 | **<0.001** |
| **Neutropenia (< 1x10^9^/L) during first 2 cycles** | 1.76 | 0.87, 3.56 | 0.11 |
| **Thrombocytopenia (< 100x10^9^/L) during first 2 cycles** | 1.71 | 0.79, 3.72 | 0.17 |
| **Lenalidomide dose** | 1.04 | 0.88, 1.23 | 0.62 |
| ^1^HR = Hazard Ratio, CI = Confidence Interval | | | |

**Table S3. Univariate analysis for progression-free survival**

| Characteristic | HR^1^ | 95% CI^1^ | p-value |
| --- | --- | --- | --- |
| **Baseline variables** | | | |
| **Gender** |  |  |  |
| Male | — | — |  |
| Female | 0.80 | 0.41, 1.59 | 0.53 |
| **Age** | 1.02 | 0.99, 1.05 | 0.32 |
| **Hemoglobin (gr/dl)** | 0.88 | 0.69, 1.13 | 0.32 |
| **Absolute neutrophil counts (x10^9^/L)** | 1.15 | 0.96, 1.37 | 0.12 |
| **Platelets (x10^9^/L)** | 1.00 | 1.00, 1.00 | **0.050** |
| **Bone marrow blasts (%)** | 1.00 | 0.90, 1.12 | 0.94 |
| **Megakaryocytic Dysplasia** | 0.83 | 0.39, 1.76 | 0.62 |
| **5q isolated** | 0.90 | 0.32, 2.52 | 0.83 |
| **IPSS-R score(1)** |  |  |  |
| Very Low/Low | — | — |  |
| Intermediate/High | 1.17 | 0.59, 2.31 | 0.65 |
| **WHO 2016 diagnosis(2)** |  |  |  |
| MDS with isolated del(5q) | — | — |  |
| MDS-EB-1/MDS-EB-2 | 1.07 | 0.42, 2.72 | 0.88 |
| **Erythropoietin (mU/mL)** | 1.00 | 1.00, 1.00 | 0.19 |
| **Mean corpuscular volume (fL)** | 1.02 | 1.00, 1.03 | **0.047** |
| **Lactate dehydrogenase (U/L)** | 1.00 | 1.00, 1.00 | 0.14 |
| **Creatinine(mg/dL)** | 2.24 | 0.92, 5.41 | 0.074 |
| **Red Blood Cells Transfusion burden** |  |  |  |
| <4 units/8 weeks | — | — |  |
| >4 units/8 weeks | 1.69 | 0.92, 3.09 | 0.089 |
| **Post-treatment variables** | | | |
| **Cytogenetic Response(3)** |  |  |  |
| No response | — | — |  |
| Partial | 0.83 | 0.37, 1.89 | 0.66 |
| Complete | 0.42 | 0.19, 0.93 | **0.033** |
| **Erythroid Response(3)** | 0.59 | 0.28, 1.24 | 0.16 |
| **Transfusion independence** | 0.53 | 0.27, 1.04 | 0.064 |
| **Neutropenia (< 1x10^9^/L) during first 2 cycles** | 1.97 | 1.04, 3.73 | **0.037** |
| **Thrombocytopenia (< 100x10^9^/L) during first 2 cycles** | 1.05 | 0.53, 2.09 | 0.88 |
| **Lenalidomide dose** | 1.11 | 0.95, 1.30 | 0.18 |
| ^1^HR = Hazard Ratio, CI = Confidence Interval | | | |

**Figure S1. Overall probability of response to lenalidomide duration.**


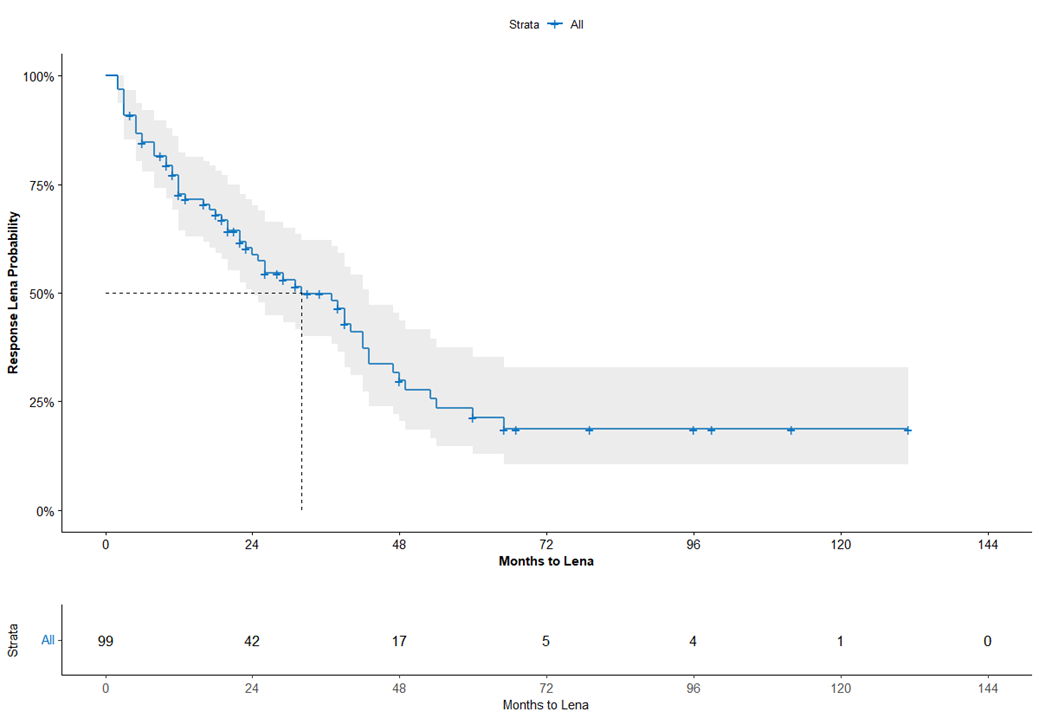


Kaplan-Meier curve shows response duration to lenalidomide. With a median response duration time of 32 months (24 - 43), the probability of being still responsive to lenalidomide was 30% (20- 44) at 48 months. Numbers at risk are indicated below the curve.

**References**

1. Greenberg PL, Tuechler H, Schanz J, Sanz G, Garcia-Manero G, Solé F, et al. Revised international prognostic scoring system for myelodysplastic syndromes. Blood. 2012;120(12):2454-65.

2. Arber DA, Orazi A, Hasserjian R, Thiele J, Borowitz MJ, Le Beau MM, et al. The 2016 revision to the World Health Organization classification of myeloid neoplasms and acute leukemia. Blood. 2016;127(20):2391-405.

3. Cheson BD, Greenberg PL, Bennett JM, Lowenberg B, Wijermans PW, Nimer SD, et al. Clinical application and proposal for modification of the International Working Group (IWG) response criteria in myelodysplasia. Blood. 2006;108(2):419-25.
